# E2F1-regulated long non-coding RNA RAD51-AS1 promotes cell cycle progression, inhibits apoptosis and predicts poor prognosis in epithelial ovarian cancer

**DOI:** 10.1038/s41598-017-04736-z

**Published:** 2017-06-30

**Authors:** Xiaodan Zhang, Guoping Liu, Junjun Qiu, Ning Zhang, Jingxin Ding, Keqin Hua

**Affiliations:** 1 Department of Gynecology, Obstetrics and Gynecology Hospital of Fudan University, Shanghai, China; 2 Shanghai Medical College, Fudan University, Shanghai, China; 3 Shanghai Key Laboratory of Female Reproductive Endocrine-Related Diseases, Fudan University, Shanghai, China; 4 Institutes of Brain Science and State Key Laboratory of Medical Neurobiology, Fudan University, Shanghai, China

## Abstract

Long non-coding RNA RAD51 antisense RNA 1 (RAD51-AS1, also known as TODRA) has been shown to be down-regulated by E2F1, a key cell cycle and apoptosis regulator, in breast cancer. Little is known regarding the role of RAD51-AS1 in disease. Here, we investigate the role of RAD51-AS1 in epithelial ovarian cancer (EOC). Using luciferase reporter and chromatin immunoprecipitation experiments, we verified RAD51-AS1 as a target of E2F1 under negative regulation in EOC. We then examined RAD51-AS1 expression in EOC samples using *in situ* hybridization (ISH). RAD51-AS1 was localized to the nucleus and found to be a critical marker for clinical features that significantly correlated with poor survival in EOC patients. RAD51-AS1 was also an independent prognostic factor for EOC. Overexpression of RAD51-AS1 promoted EOC cell proliferation, while silencing of RAD51-AS1 inhibited EOC cell proliferation, delayed cell cycle progression and promoted apoptosis *in vitro* and *in vivo*. RAD51-AS1 may participate in carcinogenesis via regulation of p53 and p53-related genes. Our study highlights the role of RAD51-AS1 as a prognostic marker of EOC. Based on its regulation of the tumor suppressor p53, RAD51-AS1-based therapy may represent a viable therapeutic option for EOC in the near future.

## Introduction

Epithelial ovarian cancer (EOC) accounts for over 90% of all ovarian malignancies and is the most lethal gynecologic malignancy^1^. This fact is largely due to the advanced stage and frequent metastasis at diagnosis in most patients^2^. Despite advances in chemotherapy and surgery, the prognosis of advanced-stage EOC patients remains poor, and ovarian cancer continues to have a high fatality rate. Therefore, studies investigating the molecular abnormalities and pathogenesis of EOC are indispensable. Finding optimal prognostic markers and therapeutic agents for ovarian cancer patients is necessary to improve disease outcomes.

In humans, long non-coding RNAs (lncRNAs), >200 bp in length, have drawn increasing attention for their extensive function in cancer biology^3, 4^. Emerging data show that the dysregulated expression of lncRNAs contributes to carcinogenesis through the disruption of normal cell processes, quintessentially by facilitating the epigenetic repression of downstream target genes^4^. Furthermore, lncRNAs, such as HOTAIR, MALAT1 and ANRIL, have been identified as prognostic biomarkers^5–8^, and the value of lncRNAs in cancer therapy has been the focus of intensive studies^9, 10^. Determining the lncRNAs involved in EOC progression may result in a better understanding of the molecular mechanisms of cancer development and may facilitate the identification of biomarkers or therapeutic targets to improve patient outcomes.

Recently, a novel lncRNA, RAD51 antisense RNA 1 (RAD51-AS1, also known as TODRA), located on 15q15.1 and consisting of two exons, captured our attention. RAD51-AS1 possesses a conserved E2F1 binding site in the promoter region and has previously been identified as a target gene of E2F1 in breast cancer by Gazy *et al*.^11^. E2F1 is a member of the E2F family of transcription factors and is best known for its role in cell cycle control and regulation of apoptosis, particularly the induction of p53-dependent apoptosis^12–19^. DNA methylation of E2F1 is an independent prognostic factor in ovarian cancer^20^, and dysregulation of E2F1 target genes correlates with a significantly worse prognosis in ovarian cancer^12^. Consequently, RAD51-AS1, a target gene of E2F1, may be involved in the regulation of cell cycle or apoptosis and participate in ovarian cancer development. Because little is known regarding the function of RAD51-AS1, the main aim of this study was to elucidate the role of RAD51-AS1 in EOC progression.

## Results

### RAD51-AS1 is a target gene of E2F1

E2F1 had been shown to bind to the same motif and oppositely regulate RAD51 and RAD51-AS1^11^, and is prefer to bind to proximal promoters^21^. Consequently, we performed CHIP of E2F1 using SKOV3.ip and HO8910 cell lines followed by PCR. We designed ten pairs of primers, which cover 3 Kb upstream of Open Reading Frame of RAD51-AS1, and we found the putative binding site of E2F1 might located at 564–825 bp upstream of the Open Reading Frame of RAD51-AS1, a sequence in RAD51 intron 1 (Supplementary Fig. S1B). We then examined the effect of E2F1 on RAD51-AS1 promoter activity in SKOV3.ip and HO8910 cells. Luciferase assays showed that E2F1 expression resulted in an approximately 40% reduction of RAD51-AS1 promoter activity (Supplementary Fig. S1A). Furthermore, 722–730 bp upstream of the Open Reading Frame of RAD51-AS1 was mutated (TTTTCCGC → AATAGGCG). Abolishing the E2F binding site restored RAD51-AS1 promoter activity back to approximately 90% (Supplementary Fig. S1A). These observations confirmed that RAD51-AS1 is directly regulated by E2F1.

### RAD51-AS1 is expressed in the nucleus and correlates with malignant clinicopathological features in EOC

**Figure 1:**
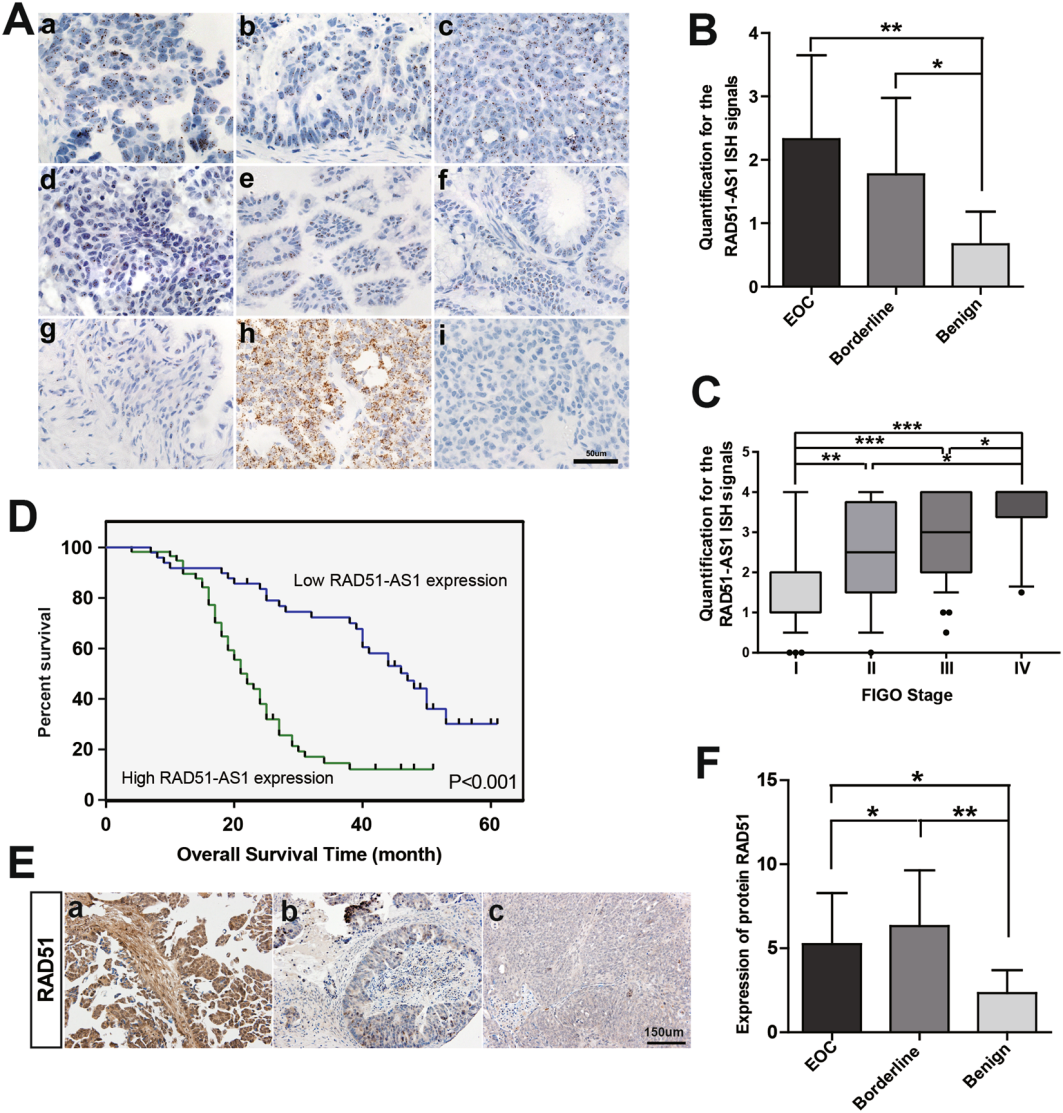
High expression of RAD51-AS1 correlated with poor prognosis in EOC. (**A**), Representative images of ISH for RAD51-AS1 in patient samples. a–d, EOC (a, serous. b, mucinous. c, endometrial. d, clear cell carcinoma). e and f, borderline ovarian tumor (e, serous. f, mucinous). g, benign ovarian tumor. h, positive control. i, negative control. ISH of a-g was performed using RAD51-AS1 target probes. ISH of h and i was performed using positive and negative probes, respectively. (**B**), Semi-quantitative analysis of ISH data of RAD51-AS1 expression in EOC (n = 129), borderline ovarian tumors (n = 22) and benign ovarian tumors (n = 12). (Mann-Whitney test) (**C**), Semi-quantitative analysis of ISH data of RAD51-AS1 expression in EOC samples of different grades. Grade I (n = 47), grade II (n = 21), grade III (n = 51) and grade IV (n = 10), (90% CI; Mann-Whitney test). (**D**), Kaplan-Meier analysis of OS was performed based on RAD51-AS1 expression levels (P < 0.001, log-rank test). (**E**), Representative images of IHC for RAD51 in patient samples. Figure 1E-a, b, c correspond to the same samples in Fig. 1A-a, b, c, respectively. (**F**), Analysis of protein RAD51 expression in EOC, borderline ovarian tumors and benign ovarian tumors (Mann-Whitney test). (*P < 0.05; **P < 0.01; ***P < 0.001).

RAD51-AS1 expression in tissue microarrays of 163 patients (129 cases of EOC, 22 cases of borderline ovarian tumors and 12 cases of benign ovarian tumors) was semi-quantitatively examined by ISH (Fig. 1A and Supplementary Fig. S2A). RAD51-AS1 was robustly expressed in the nucleus, with only very few punctate dots (one or fewer in a 40× magnification field) found outside the nucleus. Both EOC and borderline ovarian tumor tissues showed a significant increase in RAD51-AS1 expression compared with that observed in benign ovarian tissues (Fig. 1B). There were significant differences in RAD51-AS1 expression between each two grades (grade I, II, III and IV) of EOC, except between grade II and III (Fig. 1C).

Furthermore, the median value of RAD51-AS1 expression was used as a cut-off to divide the 129 EOC patients into a high-RAD51-AS1 expression group (n = 64) and a low-RAD51-AS1 expression group (n = 65). Clinicopathological features were compared between the two groups. High RAD51-AS1 expression correlated with advanced FIGO stage, histological subtype, high histological grade, number of tumors and distant metastasis (Table 1). These findings suggest that RAD51-AS1 may function in the nucleus and has a strong relationship with a more malignant ovarian cancer phenotype.

**Table 1:** Association of RAD51-AS1 expression with clinicopathological features of EOC patients.

Variable	Low RAD51-AS1 expression (n = 65)	High RAD51-AS1 expression (n = 64)	P
	n (%)	n (%)	
**Age (years)**			
<50	25 (38.5)	17 (26.6)	0.149
≥50	40 (61.5)	47 (73.4)	
**Histological subtype**			
Other	40 (61.5)	21 (32.8)	0.001
Serous	25 (38.5)	43 (67.2)	
**FIGO Stage**			
I–II	46 (70.8)	21 (32.8)	0.000
III–IV	19 (29.2)	43 (67.2)	
**Histological grade**			
Low	46 (70.8)	19 (29.7)	0.000
High	19 (29.2)	45 (70.3)	
**Tumor size (cm** ^**3**^ **)**			
<1000	44 (67.7)	45 (70.3)	0.748
≥1000	21 (32.3)	19 (29.7)	
**Number of tumors**			
<2	39 (60.0)	21 (32.8)	0.002
≥2	26 (40.0)	43 (67.2)	
**Residual tumor diameter (cm)**			
<1	55 (84.6)	45 (70.3)	0.052
≥1	10 (15.4)	19 (29.7)	
**Lymph node metastasis**			
Absent	51 (78.5)	47 (73.4)	0.504
Present	14 (21.5)	17 (26.6)	
**Distant Metastasis**			
Absent	48 (73.8)	27 (42.2)	0.000
Present	17 (26.2)	37 (57.8)	
**CA125 level (U/ml)**			
<600	58 (89.2)	52 (81.3)	0.201
≥600	7 (10.8)	12 (18.8)	
**Ascites**			
Absent	53 (81.5)	43 (67.2)	0.062
Present	12 (18.5)	21 (32.8)	

NOTE: The median value of RAD51-AS1 expression was used as a cut-off for the low/high RAD51-AS1 expression groups. Data were analyzed using the χ^2^ test. All tests were two-sided, and P < 0.05 was considered significant.

In a previous study, there was opposite regulation of RAD51-AS1 and RAD51 in breast cancer^11^, so we detected the expression of RAD51 by IHC on the tissue microarrays (Fig. 1E and Supplementary Fig. S2B). However, the result demonstrated that RAD51-AS1 expression had no correlation with RAD51 expression (P = 0.755, r = −0.026, Spearman correlation). It was found that borderline ovarian tumor had a higher expression of RAD51 than EOC, and both of them showed a significant increase in RAD51 expression compared with that observed in benign ovarian tissues (Fig. 1F). There was no significant difference in RAD51 expression among different FIGO stages of EOC (Supplementary Fig. S2C). High level of RAD51 correlated with smaller tumor size in EOC (P = 0.023). However, no statistical significance was found between RAD51 expression and other clinicopathological features of EOC patients (Supplementary Table S1).

### RAD51-AS1 overexpression confers a poor prognosis and is an independent prognostic factor in EOC patients

Excluding 23 EOC patients who were lost to follow-up, complete outcome data were obtained in 106 EOC patients (follow-up rate, 82.2%). Survival analysis showed that EOC patients with high RAD51-AS1 expression levels had a significantly decreased overall survival (OS) than those with low RAD51-AS1 expression levels (P < 0.001, Fig. 1D). In addition, multivariate Cox regression analysis revealed that FIGO stage and RAD51-AS1 expression were independent predictors of OS in EOC (P < 0.05, Table 2).

**Table 2:** Univariate and multivariate analysis of overall survival in 106 EOC patients.

Variables	Univariate analysis		Multivariate analysis					
	Overall survival (months) Mean ± SE	P	Overall survival					
			β	SE	Wald	P	Exp(β)	95%CI for Exp(β)
RAD51-AS1 expression								
Low	43.05 ± 2.51	<0.001						
High	24.60 ± 1.63		0.764	0.349	4.786	**0.029**	2.148	1.083–4.261
Age (years)								
<50	29.13 ± 2.51	0.126						
≥50	35.43 ± 2.25		−	−	−	−	−	−
Histological subtype								
Serous	40.73 ± 2.77	<0.001						
Others	26.82 ± 1.75		0.028	0.352	0.006	0.936	1.029	0.517–2.049
FIGO Stage								
I-II	44.89 ± 2.29	<0.001						
III-IV	20.85 ± 1.09		2.150	0.525	16.782	<**0.001**	8.582	3.068–24.001
Histological grade								
Low	38.95 ± 2.51	<0.001						
High	26.31 ± 1.84		0.165	0.361	0.209	0.647	1.179	0.582–2.392
Tumor size (cm^3^)								
<1000	33.54 ± 2.16	0.663						
≥1000	34.28 ± 3.17		−	−	−	−	−	−
Number of tumors								
<2	41.33 ± 2.61	<0.001						
≥2	27.10 ± 2.00		0.271	0.311	0.758	0.384	1.311	0.712–2.413
Residual tumor diameter (cm)								
<1	36.60 ± 2.10	<0.001						
≥1	22.08 ± 1.31		0.221	0.345	0.410	0.522	1.247	0.634–2.452
Lymph node metastasis								
Absent	37.21 ± 2.10	<0.001						
Present	22.34 ± 2.28		0.053	0.316	0.028	0.867	1.054	0.567–1.959
Distant Metastasis								
Absent	40.96 ± 2.34	<0.001						
Present	23.38 ± 1.80		0.513	0.435	1.393	0.238	1.671	0.712–3.919
CA125 level (U/ml)								
<600	35.12 ± 1.92	0.006						
≥600	20.58 ± 1.80		0.130	0.392	0.110	0.740	1.139	0.528–2.458
Ascites								
Absent	38.04 ± 2.19	<0.001						
Present	22.32 ± 1.72		0.278	0.359	0.598	0.439	1.320	0.653–2.668
Number of chemotherapy								
≤6	36.81 ± 2.23	0.006						
>6	26.55 ± 2.39		0.034	0.322	0.011	0.916	1.035	0.550–1.946

All tests were two-sided, and P < 0.05 was considered significant.

### RAD51-AS1 promotes EOC cell proliferation, regulates cell cycle progression and cell apoptosis

**Figure 2:**
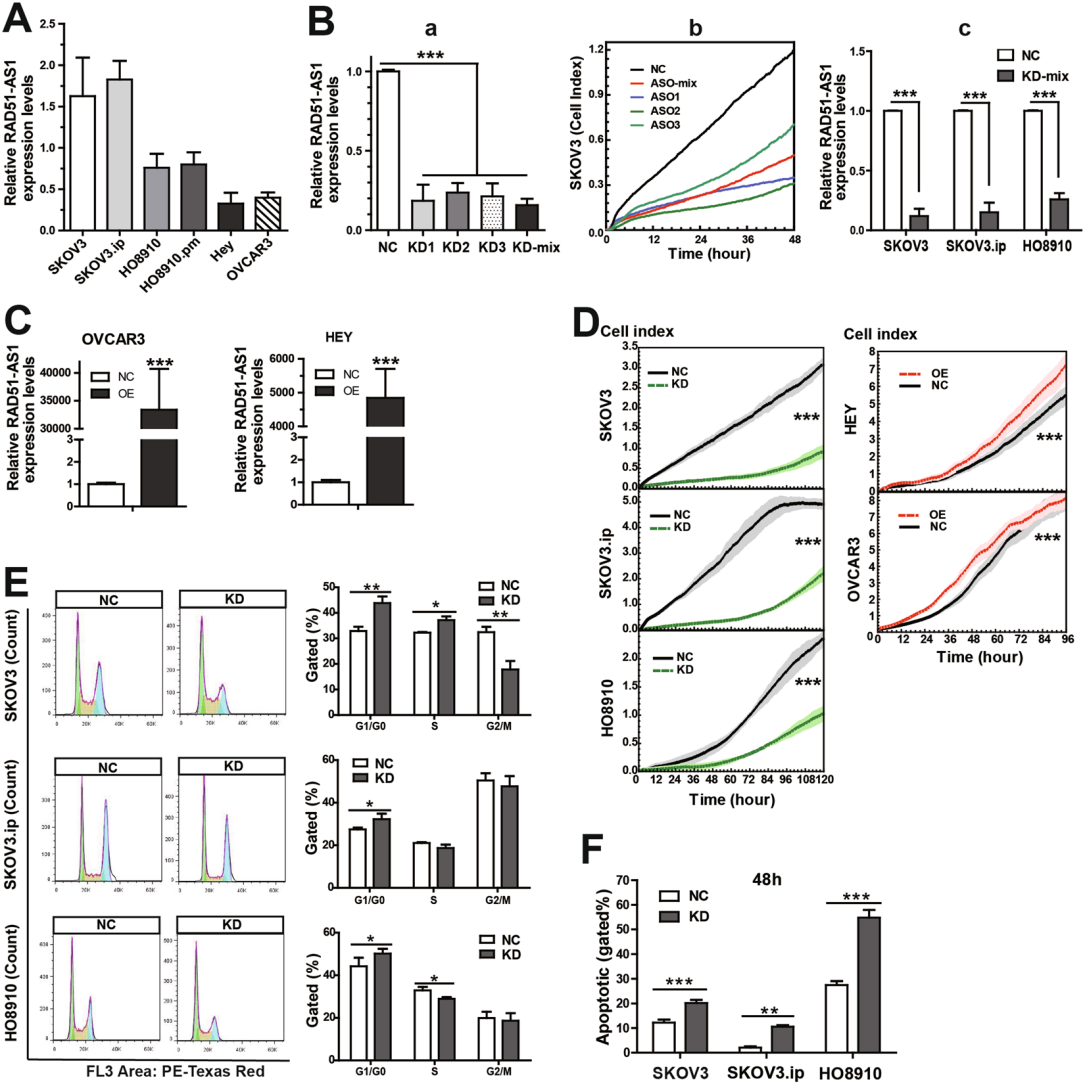
Study of the biological and functional characteristics of RAD51-AS1 *in vitro*. (**A**), Relative RAD51-AS1 expression in EOC cell lines. (**B**), a,b: QRT-PCR and proliferation assay to quantify the silencing efficiencies of RAD51-AS1 in SKOV3 cells transfected with ASO1, ASO2, ASO3 and ASO-mix. c: QRT-PCR confirmed that RAD51-AS1 was efficiently silenced by transfection with ASO-mix in SKOV3, SKOV3.ip and HO8910 cells. (**C**), The efficiency of RAD51-AS1 overexpression was confirmed by qRT‑PCR (**D**), Proliferation assays. Cell growth curves were automatically recorded in real-time. Silencing of RAD51-AS1 inhibited cell proliferation in SKOV3, SKOV3.ip and HO8910 cells. Overexpression of RAD51-AS1 increased the proliferation of Hey and OVCAR3 cells. The light-colored error bars denote the SD. (**E**), Cell cycle assays. RAD51-AS1 knockdown increased the percentage of cells in G1/G0 phase, while the effects on S and G2/M phages are not consistent among the 3 cell lines. (**F**), Apoptosis assays. RAD51-AS1 knockdown increased the percentage of apoptotic cells in SKOV3, SKOV3.ip and HO8910 cells. Each experiment was repeated in triplicate (n = 3). KD, knockdown of RAD51-AS1 expression. OE, overexpression of RAD51-AS1, NC, negative control. (The results are shown as the mean ± SD; *P < 0.05; **P < 0.01; ***P < 0.001 by Student’s t test).

We evaluated RAD51-AS1 expression in EOC cell lines (Fig. 2A), and the results indicated general expression in these cells. The highest expression levels were detected in SKOV3.ip cells and the lowest in Hey cells. SKOV3, SKOV3.ip and HO8910 cells were then chosen for knockdown experiments, while Hey and OVCAR3 were for overexpression experiments. We designed three ASOs (ASO1, ASO2 and ASO3) targeting RAD51-AS1. In this study, we used an ASO-mix, a ‘cocktail’ combination of ASOs with equimolar ASO1, ASO2 and ASO3, to reduce the off-target effects of ASOs while silencing the expression of RAD51-AS1. Their silencing efficiencies were quantified by qRT-PCR analysis (Fig. 2B-a) and proliferation assay (Fig. 2B-b). As shown in Fig. 2B-c, the ASO mix exerted a satisfactory silencing effect on RAD51-AS1 expression in SKOV3, SKOV3.ip and HO8910 cells. Additionally, the efficiency of RAD51-AS1 overexpression was confirmed by qRT‑PCR (Fig. 2C).

Thereafter, we conducted a series of functional experiments on cells in which RAD51-AS1 expression had been knocked down or overexpressed. Cell proliferation assays showed that RAD51-AS1 silencing significantly inhibited the proliferation of SKOV3, SKOV3.ip and HO8910 cells, and RAD51-AS1 overexpression increased the proliferation of Hey and OVCAR3 cells (Fig. 2D). Cell cycle assays showed that RAD51-AS1 knockdown increased the cells in G1/G0 phase in all 3 cell lines (Fig. 2E). Changes in S-phase were cell line-specific, with SKOV3 showing an increase, SKOV3.ip showing no change, and H08910 showing a decrease. Decrease change in G2/M was only shown in the SKOV3 cells, and not the others. In addition, knockdown of RAD51-AS1 remarkably increased the percentage of apoptotic cells (Fig. 2F and Supplementary Fig. S3D). These results indicate that RAD51-AS1 promotes proliferation *in vitro*.

### RAD51-AS1 knockdown inhibits cell migration and invasion *in vitro*

**Figure 3:**
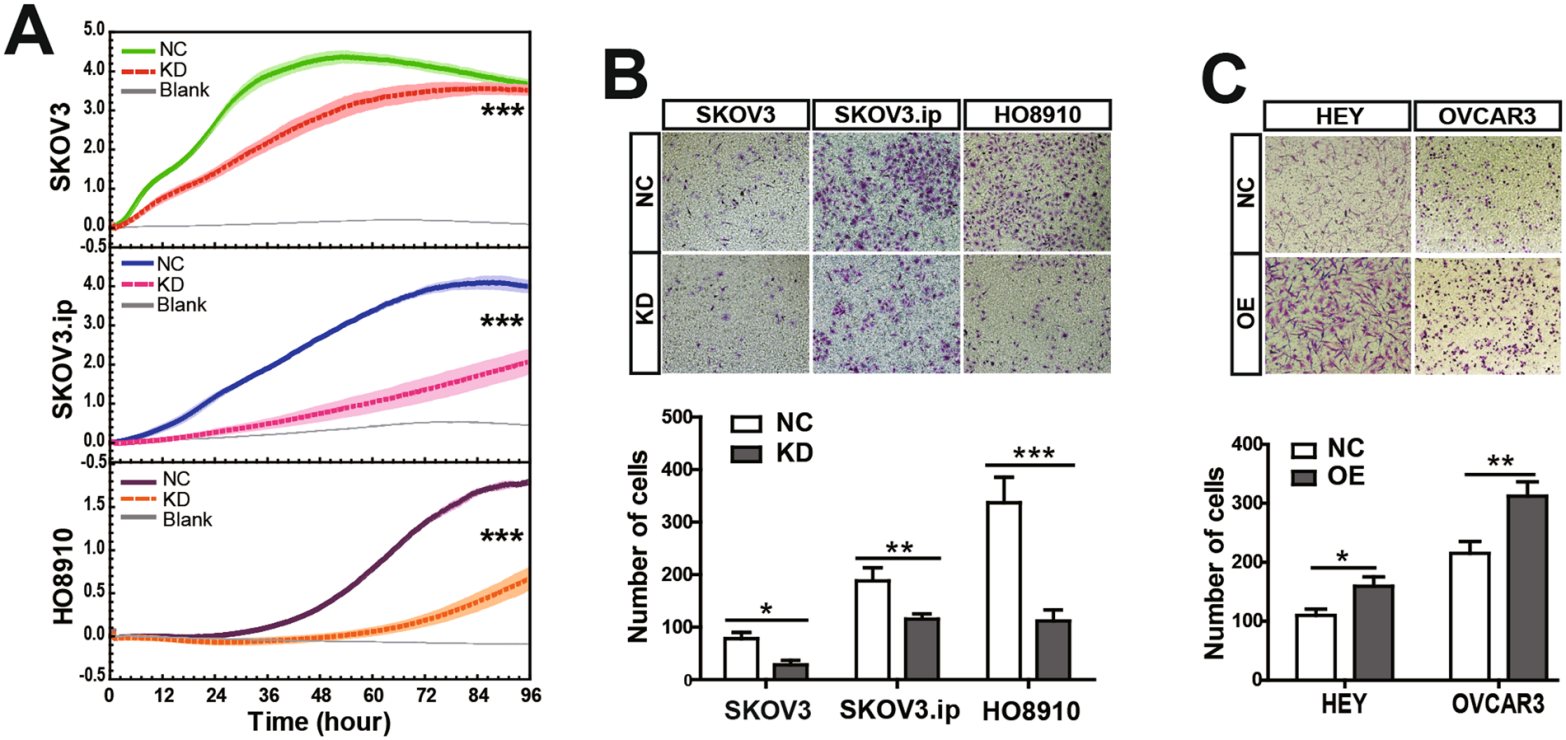
Study of functional characteristics of RAD51-AS1 in cell migration and invasion. (**A**), Migration assays. The real-time cell indexes indicated that RAD51-AS1 knockdown greatly decreased cell migration capacity compared with the NC cells. Blank control was performed with the bottom chamber containing RPMI 1640 but without FBS as a chemo-attractant. The light-colored error bars denote the SD. (**B**), Transwell invasion assays. RAD51-AS1 knockdown significantly decreased the invasive capacity of SKOV3, SKOV3.ip and HO8910 cells. (**C**) Overexpression of RAD51-AS1 increased cell invasion capacity of Hey and OVCAR3 cells. Each experiment was repeated in triplicate (n = 3). (The results are shown as the mean ± SD; *P < 0.05; **P < 0.01; ***P < 0.001 by Student’s t test).

As RAD51-AS1 correlates with distant metastasis, we performed migration and invasion experiments to explore the function of RAD51-AS1 in EOC metastasis. The migration assay showed that the cell indexes in RAD51-AS1 knockdown group were greatly lower than in the control group (Fig. 3A). The Matrigel invasion assay indicated that the suppression of RAD51-AS1 expression significantly inhibited the invasive ability of SKOV3, SKOV3.ip and HO8910 cells (Fig. 3B). What’s more, overexpression of RAD51-AS1 increased cell invasion in Hey and OVCAR3 cells (Fig. 3C).

In order to normalize the potential effect of cell proliferation, Mitomycin C was added to the culture media to make the cells uniformly arrested. The results showed that all migration cell indexes reduced after adding Mitomycin C than those observed without Mitomycin C. Knockdown of RAD51-AS1 still inhibited cell migration (Supplementary Fig. S3A) and invasion of SKOV3, SKOV3.ip and HO8910 cells (Supplementary Fig. S3B), while overexpression of RAD51-AS1 only increased cell invasion capacity of the OVCAR3 cells, not in the Hey cells (Supplementary Fig. S3C). Overall, these findings illustrated that RAD51-AS1 promotes EOC cell migration and invasion *in vitro*.

### p53 and p53-related genes are key downstream mediators of RAD51-AS1 regulation in EOC

**Figure 4:**
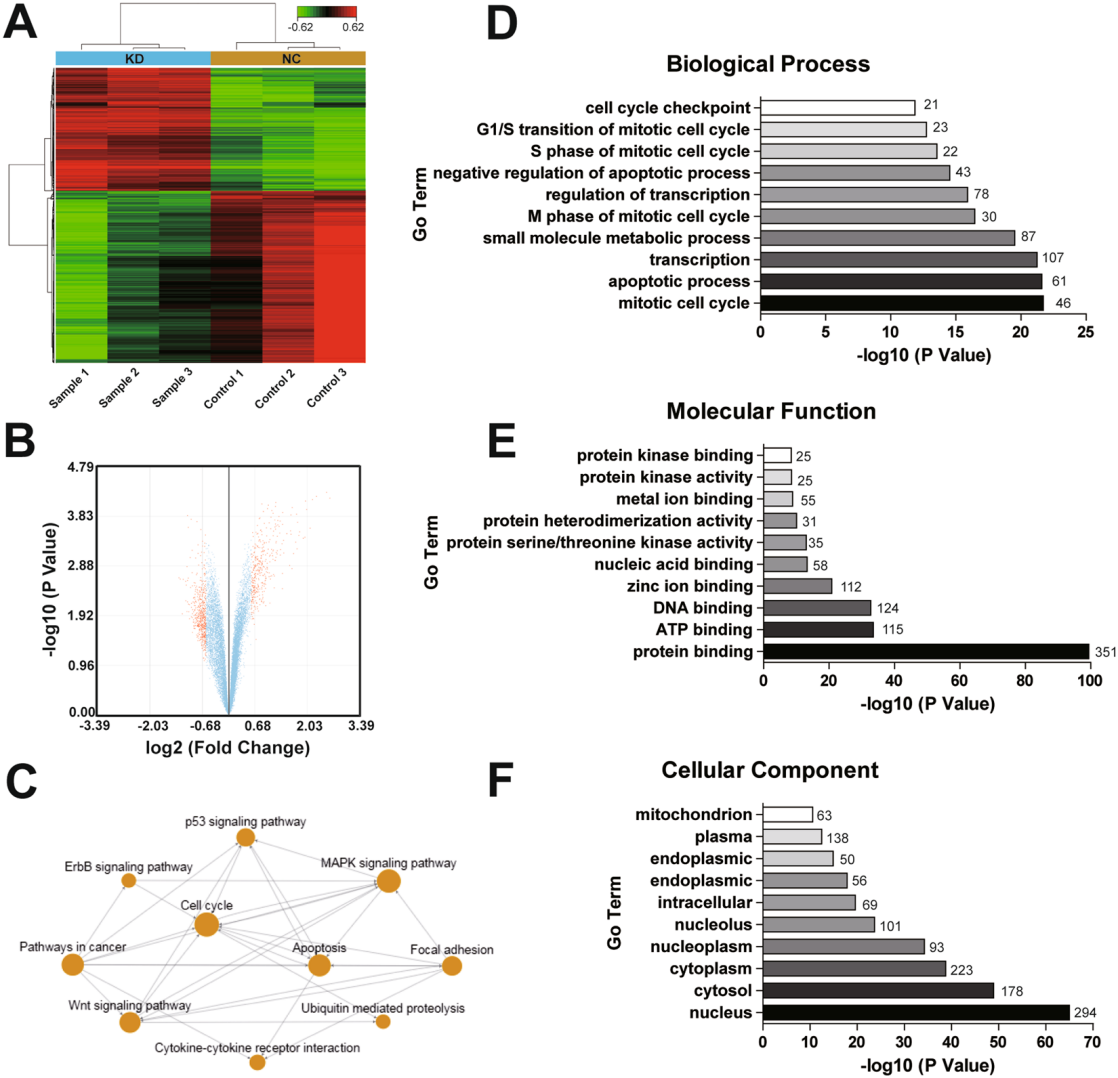
RAD51-AS1 knockdown regulates gene expression in SKOV3.ip cells. (**A**), Hierarchical clustering of the genes differentially expressed in SKOV3.ip cells 24 hours after RAD51-AS1 silencing. Each column represents one sample and each row represents one gene. The color scale at the top of the figure illustrates the relative expression of a gene across all of the samples, with red indicating high expression and green indicating low expression. (**B**), Volcano plot shows that some genes were differentially expressed after RAD51-AS1 knockdown. (**C**), Analysis of pathway relation network. The interactions in the KEGG Database were used to construct the interaction network between pathways, and the top ten pathways after silencing RAD51-AS1 are shown. (**D**,**E** and **F**), The significant analysis of Gene Ontology (GO) on three aspects (D, biological process analysis; **E**, molecular function analysis; F, cellular component analysis) were performed with parameter settings of P = 0.05, FDR = 0.05, and the top ten terms are listed.

To further identify the mechanisms and downstream regulators of RAD51-AS1, we performed a microarray analysis. Three SKOV3.ip RAD51-AS1-knockdown samples and three control samples were used. After silencing RAD51-AS1 expression, we identified 962 differentially expressed coding genes (Q value < 0.05, Fold change ≥1.5, 401 up-regulated and 561 down-regulated), which are represented in the heat map (Fig. 4A) and volcano plot (Fig. 4B).

Then, the significant GO analysis was performed (P = 0.05, FDR = 0.05), and the top ten GO terms are listed. In biological process analysis, mitotic cell cycle changed most significantly, followed by apoptotic process and transcription (with 46, 61 and 107 differentially expressed gene counts in GO, respectively) (Fig. 4D). It is interesting to note that these findings are consistent with our original hypothesis. In the molecular function analysis, protein binding presented 351 differentially expressed gene counts in GO, far more than ATP binding and DNA binding (Fig. 4E). In the cellular component analysis, the nucleus was the most represented component with an apparent advantage over others (with 294 differentially expressed gene counts in GO), consistent with our ISH results on RAD51-AS1 expression in the nucleus (Fig. 4F). These results suggest that RAD51-AS1 mainly regulates cell cycle and apoptosis progression in EOC. Expressed in the nucleus, RAD51-AS1 may function through protein binding to exert downstream effects.

**Figure 5:**
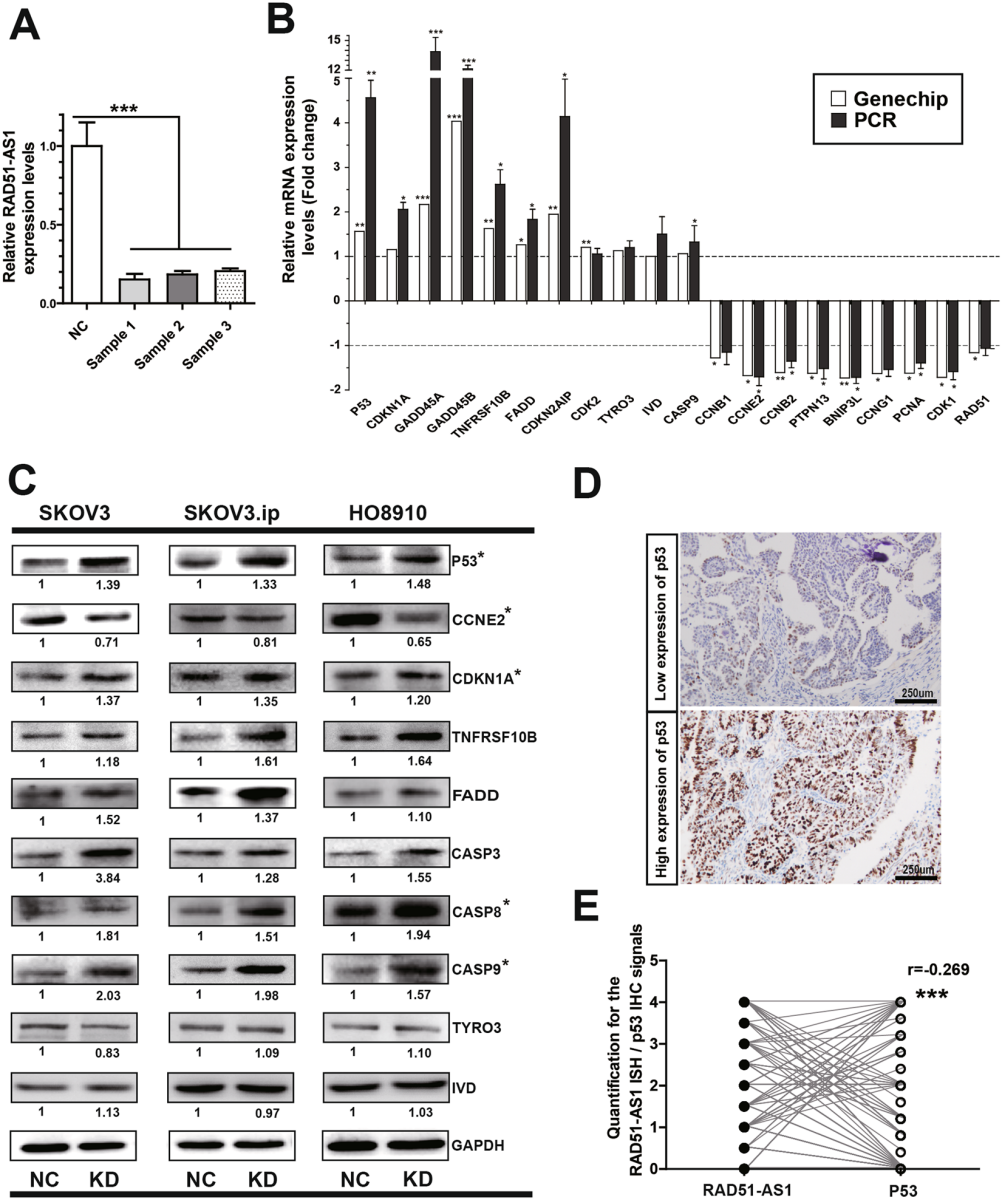
RAD51-AS1 silencing regulates the expression of p53 and p53-related genes. (**A**), The silencing efficiency of RAD51-AS1 in microarray samples was validated by qRT-PCR. (**B**), The mRNA levels of p53-related genes in SKOV3.ip cells were detected by qRT-PCR after RAD51-AS1 knockdown. The results confirm the microarray data, and the mRNA level of predicted RAD51-AS1 target genes (TYRO3 and IVD) was not affected. (**C**), The protein levels of p53-related genes were detected by Western blotting after RAD51-AS1 knockdown. No changes were observed in the protein levels of TYRO3 and IVD. (**D**), Representative p53 IHC images in EOC patient samples. (**E**), The correlation between p53 and RAD51-AS1 expression in patient samples (Spearman Correlation analysis, P < 0.001, r = −0.269). All results are shown as the mean ± SD and are representative of three independent experiments. (*P < 0.05; **P < 0.01; ***P < 0.001 by Student’s t test).

Next, a pathway relation network was developed based on the KEGG database to help define the synergistic effects of the most important pathways (Fig. 4C). The result highlighted that the p53 pathway was closely involved in those changes. Accordingly, we focused on genes in the p53 pathway, particularly p53-related genes associated with cell cycle and apoptosis, in the downstream regulation of RAD51-AS1. We used qRT-PCR to confirm the interference efficiency of RAD51-AS1 in the microarray (Fig. 5A) and detected the transcription levels of p53 and p53-related genes (Fig. 5B). We determined that the mRNA of p53 increased 4.56-fold after silencing RAD51-AS1 (1.56-fold change in the microarray). The expression of p53-related genes involved in cell cycle or apoptosis, such as CDKN1A, TNFRSF10B, FADD, CASP3, CASP8 and CASP9 was higher, while CCNE2 levels were lower in the RAD51-AS1-knockdown group compared with the control group. Their expression confirmed our microarray results, thus ensuring the reliability of the HTA2.0 array. In addition, Western blot analysis was used to examine the protein levels of these genes. Changes of P53, CCNE2, CDKN1A, CASP8 and CASP9 were found statistically significant (Fig. 5C). Finally, we evaluated p53 expression in patient samples (Fig. 5D). The correlation analysis demonstrated that p53 expression negatively correlated with RAD51-AS1 expression (P < 0.001, r = −0.269, Fig. 5E). These results reinforce the possibility that p53 and p53-related genes are key downstream mediators of RAD51-AS1.

### RAD51-AS1 knockdown impairs tumor growth *in vivo*

**Figure 6:**
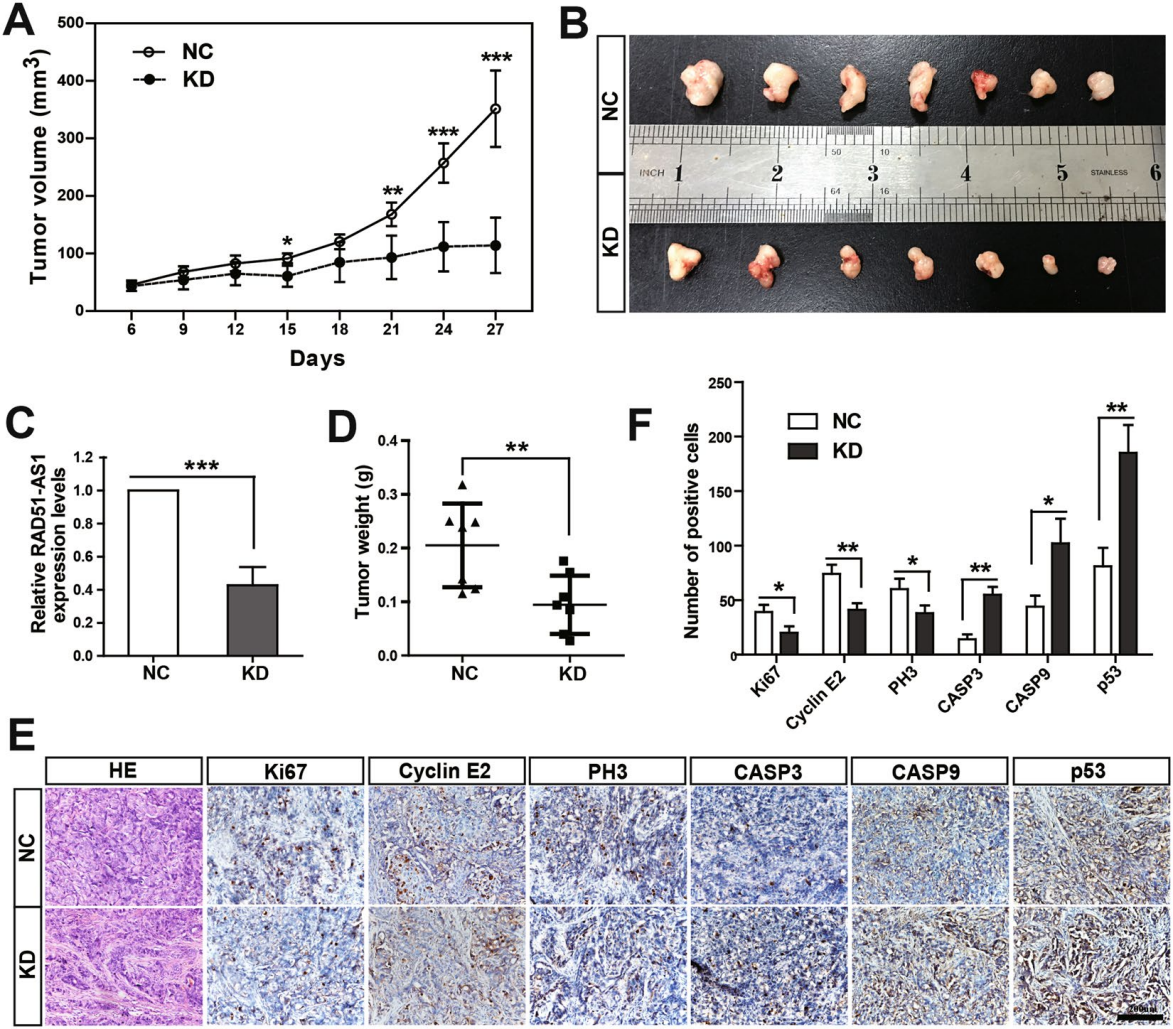
RAD51-AS1 knockdown impairs tumor growth *in vivo*. (**A**), Growth curves of xenograft tumors. Tumor growth in the RAD51-AS1-knockdown group was substantially slower than that in control group, and the gap between the two groups increased over time. (**B**), Visual comparison of the tumors in the RAD51-AS1-knockdown group and the control group. (**C**) The efficiency of RAD51-AS1 silencing *in vivo* was validated by qRT-PCR. (**D**), The tumor weight in the RAD51-AS1 knockdown group was significantly decreased compared with the controls. (**E**), Xenograft tumor tissue sections were stained with hematoxylin-eosin-saffron (HE). Representative images of immunohistochemical staining for Ki67, CCNE2, PH3, CASP3, CASP9, p53 are shown. (**F**). Statistical analysis of the IHC experiments. (*P < 0.05; **P < 0.01; ***P < 0.001 with Student’s t test).

To evaluate the effect of RAD51-AS1 *in vivo*, xenograft tumors were established in nude mice. As shown in Fig. 6A, tumor growth in the RAD51-AS1 knockdown group was substantially slower than that in the control group, and the gap between the two groups increased with time. After the mice were sacrificed, we performed qRT-PCR to confirm the silencing of RAD51-AS1 *in vivo* (Fig. 6C). By visual comparison, it was obvious that tumors in the RAD51-AS1-knockdown group were substantially smaller than those in the control group (Fig. 6B). In addition, the tumor weight in the RAD51-AS1-knockdown group decreased by almost 50% compared with the control group (Fig. 6D). These results indicate that silencing of RAD51-AS1 impairs tumor growth *in vivo*.

Based on the findings *in vitro*, we then detected certain key regulatory factors by IHC to further clarify the function of RAD51-AS1 *in vivo* (Fig. 6E–F). As expected, p53 expression was increased following RAD51-AS1 silencing. In addition, the expression of the proliferation marker Ki67 was significantly lower in RAD51-AS1-knockdown tumors. Similarly, PH3 (an M-phase marker of the cell cycle) and CCNE2 (a core component of cell cycle machinery, particularly the G1/S phase transition) presented with lower expression levels, consistent with the cell cycle assay. In contrast, the expression levels of apoptotic factors (CASP3 and CASP9) were significantly higher in RAD51-AS1-knockdown tumors than in the control ones. Taken together, these results support our findings *in vitro* and suggest that RAD51-AS1 promotes cell proliferation and cell cycle progression and inhibits cell apoptosis *in vivo*.

## Discussion

Numerous studies have revealed that lncRNAs have distinct biological functions and essential roles in tumorigenesis^22^. LncRNAs, such as HOTAIR, ANRIL and MALAT1, have been suggested as potential prognostic biomarkers and therapeutic targets in cancers^5–8^. Therefore, a better understanding of lncRNAs may help in the identification of new biomarkers and effective therapeutic strategies that can improve patient outcomes. RAD51-AS1 is a novel lncRNA that is transcribed 69 bp upstream of RAD51, in the opposite direction. Herein, we determined that the transcription factor E2F1, a key regulator of cell cycle and apoptosis^13–19, 23^, negatively regulated RAD51-AS1 expression in EOC cell lines by binding its promoter. These findings are consistent with a previous study in breast cancer^11^.

In breast cancer, there was opposite regulation of RAD51-AS1 and RAD51 expression, and higher RAD51-AS1/lower RAD51 expression was associated with a less aggressive histological tumor phenotype^11^. However, in our study, the correlation analysis demonstrated that RAD51-AS1 expression has no correlation with RAD51 expression in EOC. Moreover, we found that RAD51-AS1 expression correlated with a more malignant phenotype and a poorer prognosis of EOC. RAD51-AS1 was an independent prognostic indicator for EOC patients. These clinical findings enhance the potential value of RAD51-AS1 as a prognostic marker for EOC.

In support of the hypothesis posed at the beginning of this study, *in vitro* and *in vivo* experiments provide evidence that RAD51-AS1 is involved in the regulation of cell cycle or apoptosis and plays a role in promoting cell proliferation in EOC. The microarray results reinforce our findings in cytobiology experiments. Additionally, RAD51-AS1 also regulates cell migration and invasion in EOC cells.

We found there was enrichment for genes expressed in the nucleus through GO analysis. Using ISH, RAD51-AS1 was found to be strongly expressed in the nucleus, where it most likely functions through binding to proteins. Mechanistically, the most well-known lncRNAs regulate transcription through interactions with protein, DNA, or other cellular macromolecules^24^. In addition, recent studies have shown that lncRNAs expressed in the nucleus mostly regulate cell processes by facilitating the epigenetic repression of downstream genes^4, 25^. For instance, ANRIL, HOTAIR, H19 and XIST all play a repressive function by coupling with histone modifying or chromatin remodeling protein complexes^26–30^. Thus, we speculated that RAD51-AS1 may function through binding to proteins, such as transcription factors, to achieve downstream effects; some key genes might be repressed by RAD51-AS1.

Genome browser UCSC hg19 (http://genome.ucsc.edu/) was used to get DNA sequences. Target genes under cis-regulatory control were defined as genes whose transcription was regulated by lncRNAs in nearby genomic locations (≤10 kbp upstream or downstream)^31^. Based on this, we identified two predicted target genes of RAD51-AS1: Tyro3 and IVD. However, neither the mRNA nor protein levels of these molecules changed after silencing RAD51-AS1 expression. Then, the p53 pathway highlighted by KEGG pathway analysis stimulated our interest. p53 activation can cause cell cycle arrest and apoptosis^32, 33^, which is the exact phenotype observed upon RAD51-AS1 silencing. We found that both mRNA and protein levels of p53 were elevated by RAD51-AS1 silencing. Furthermore, the expression of RAD51-AS1 and p53 showed reverse correlation in patient tissues, raising the possibility that p53 is a key downstream gene repressed by RAD51-AS1. In general, elevated levels of p53 protein will in turn induce CDKN1A transcription and lead to cell cycle arrest at the G1 phase^34, 35^. As expected, we detected elevated CDKN1A after silencing RAD51-AS1. In addition, RAD51-AS1 silencing activates apoptotic regulators associated with p53 up-regulation, which may explain the pro-apoptotic effect of RAD51-AS1 silencing. These findings not only further illustrate that RAD51-AS1 regulates cell cycle and apoptosis but also support that p53 and p53-related genes are key downstream mediators of RAD51-AS1. Their dysreg8ulation may partially explain the involvement of RAD51-AS1 in EOC development.

As the number of well-described lncRNAs grows and along with the development of RNAi-based therapeutics^36^, the value of lncRNAs in cancer therapy has been attracting increasing attention^9, 10, 37^. Based on the reverse regulation of the tumor suppressor p53, one might anticipate that the inhibition of RAD51-AS1 might have a therapeutic effect by restoring the expression of p53 and p53-related genes. However, it is beyond the scope of this study to examine the direct target genes of RAD51-AS1. Therefore, future studies of the detailed mechanisms of RAD51-AS1 are needed. In addition, studies with larger samples are required to enhance the feasibility and reliability of RAD51-AS1 as a prognostic biomarker for EOC.

## Materials and Methods

### Cell lines and cell culture

The human ovarian cancer cell lines (SKOV3, SKOV3.ip, HO8910 and HO8910-PM) were provided by the University of Texas MD Anderson Cancer Center (Houston, TX, USA) and were authenticated by Short Tandem Repeat (STR) profiling. The human ovarian cancer cell lines Hey and OVCAR3 were purchased from the American Type Culture Collection (ATCC, Manassas, VA, USA). All cells were cultured in RPMI 1640 (Gibco BRL, Gaithersburg, MD, USA) containing 10% fetal bovine serum (FBS; Gibco) with 100 units/ml penicillin and 100 mg/ml streptomycin in a 5% CO_2_ humidified incubator at 37 °C. Mitomycin C was purchased from MedChem Express (China, HY-13316). In the migration assay and transwell invasion assay, the cells were mitotically inactivated by treating with mitomycin C (10 µg/mL, 2 h).

### Tissue microarray and patient data

This study was approved by the ethics committee of the Obstetrics and Gynecology Hospital of Fudan University (No. [2015]33) and complies with the REMARK guidelines for biomarker studies^38^. All paraffin-embedded tissue samples were obtained from the Tissue Bank of the Obstetrics and Gynecology Hospital of Fudan University. This study included 163 patients, who underwent surgery in the Obstetrics and Gynecology Hospital of Fudan University between January 2009 and December 2011. Patients with two or more different malignancies and those who had received chemotherapy, preoperative radiotherapy, or hormonal therapy were excluded from the study. Tissue microarrays were manufactured. For each tissue sample, we collected tissues from two different areas to reduce the influence of tumor heterogeneity and to be more representative of the tumor. Clinical and histopathologic features were retrieved by reviewing medical charts and pathology reports. The outcomes of each patient were retrieved from the Follow-up Database in our hospital. Informed consent was obtained from all of the patients, according to the principles of the Declaration of Helsinki.

### *In situ* hybridization (ISH)

Paraffin sections of the tissue microarrays were analyzed according to a protocol developed by Advanced Cell Diagnostics^39^. ISH was performed using the RNAscope® 2.0 Assay (ACD, Inc. Catalog No. 320497) and HybEZ™ Hybridization System (ACD, Inc. 110 VAC, Cat. no. 310010). RAD51-AS1 target probes and positive and negative control probes were designed and purchased from ACD. Technical and slide quality control was certified using a positive control probe targeting the common housekeeping gene PPIB and a negative control probe targeting the bacterial gene DapB (PPIB > 2 and DapB < 1). Finally, slides were imaged using an Olympus Dual-CCD microscope digital camera, and semi-quantitative scores were obtained by estimating the number of punctate dots. See Supplementary Materials and Methods for details.

### Quantitative real-time PCR

Statistical analyses of the results were performed using the 2^−ΔΔCt^ relative quantification method. See Supplementary Materials and Methods for details.

### Chromatin immunoprecipitation (ChIP) and PCR

ChIP assays were performed using the Pierce Agarose CHIP Kit (Thermo, Prod# 26156) according to the manufacturer’s protocol. An E2F1 antibody (Santa Cruz, sc-193X) was used to precipitate DNA fragments. PCR was performed using Premix Taq^TM^ (TaKaRa, RR900Q). The ChIP-PCR primers and details see Supplementary Materials and Methods.

### Luciferase reporter experiments

Transfection was performed using Lipofectamine 3000 (Invitrogen Inc.). Luciferase assays were performed using the Dual-Luciferase Reporter Assay System (Promega) following the manufacturer’s instructions. Plasmid information is described in the Supplementary Materials and Methods section. Luciferase activity was measured on a luminometer (Berthold Technologies).

### RNA interference and RAF51-AS1 overexpression

We used 6 siRNAs targeting RAD51-AS1, but none knocked down RAD51-AS1 expression by more than 50%. Therefore, three antisense oligonucleotides (ASO) targeting RAD51-AS1 and a negative control (NC) were designed and synthesized by Ribobio (Guangzhou, China). Cells were transfected with 50 nM RAD51-AS1-targeting ASO or 50 nM NC using Lipofectamine 3000 (Invitrogen). Silencing efficiency was evaluated by qRT-PCR. Each ASO had a satisfactory interference effect on RAD51-AS1 expression. In this study, we used an ASO-mix (a ‘cocktail’ combination of ASOs with equimolar concentrations of ASO1, ASO2 and ASO3) to reduce off-target effects. The target sequences of RAD51-AS1-targeting ASOs are represented in the following sequences: ASO1-TCCGCGAGTTCTCACCATCG, ASO2-TCCTAGCTACTCGAAAGGCT, and ASO3-GCATGGAAACGAACTACATG. Additionally, the lentiviruses expressing the RAD51-AS1 sequence (RAD51-AS1‑overexpression, OE) and the negative control lentivirus (NC) were constructed by Hongli Co. (Shanghai, China).

### Proliferation and migration assays

Twenty-four hours after transfection, cells were seeded at 5 × 10^3^ cells/well in cell culture E-Plate 16 (ACEA Biosciences Inc., USA) for proliferation assays and at 2 × 10^4^ cells/well in cell culture CIM-Plate 16 (ACEA Biosciences Inc., USA) for migration assays according to the manufacturer’s instructions. The plates were incubated at 37 °C with 5% CO_2_. The cell growth and migration curves were automatically recorded on the xCELLigence RTCA System (Roche, USA) in real-time. The cell index of the proliferation and migration assays was followed for 4–5 days.

### Flow cytometric analysis of apoptosis and cell cycle

Cells were harvested 48 hours after transfection. For apoptosis analysis, the transfected cells were stained using the Dead Cell Apoptosis Kit with Annexin V Alexa Fluor 488 and propidium iodide (PI) (Invitrogen, catalog number V13241) according to the manufacturer’s recommendations. For cell cycle analysis, after fixing and permeabilizing the transfected cells, 0.5 ml of PI/RNase Staining Buffer (BD Biosciences, USA, catalog number 550825) was used per sample (1 × 10^6^ cells) and incubated for 30 minutes at room temperature. Thereafter, the cell apoptosis ratio and the percentage of cells in each phase of cell cycle were measured using a FACStation (FV500, Beckman Coulter, Brea, CA, USA). The results were analyzed using FlowJo 7.6.2 software.

### Transwell invasion assay

Cells were harvested 24 hours after transfection. Cell invasion was evaluated using the Transwell invasion assay with inserts of 8-µm pore size (Coring Costar), as previously described^7^.

### Microarray assay

Twenty-four hours after transfection of 50 nM RAD51-AS1-ASOs or NC, total RNA from SKOV3.ip cells was extracted using TRIzol reagent (Life Technologies). Then, the Affymetrix Human Transcriptome Array 2.0 (HTA 2.0) was used to compare the transcriptome profiling of RAD51-AS1 knockdown (KD) and control samples. Differentially expressed genes, Gene Ontology (GO) and KEGG pathway analyses were used to investigate the functional significance of the molecular changes. The Gene Expression Omnibus accession numbers are (GSE89374).

### Western blot analysis

Western blot analysis was performed as previously described^7^. The antibodies used are described in the Supplementary Materials and Methods section. The quantitative protein analysis was performed using ImageJ software.

### Xenograft tumors in nude mice

All animal studies were approved by the Institutional Animal Care and Use Committee of the Fudan University (Approval number: 20150595A186). A total of 5 × 10^6^ SKOV3.ip cells were subcutaneously injected into sixteen female athymic Balb/c nude mice (aged 4 to 6 weeks, weighing 14–18 g) purchased from Department of Laboratory Animal, Fudan University. All of the oligonucleotides for RAD51-AS1 knockdown or NC used *in vivo* were as described above and synthesized without endotoxin by Ribobio (Guangzhou, China). Experiments were performed according to the Institutional Guidelines. For details, see Supplementary Materials and Methods.

### Immunohistochemical (IHC) analysis

Tissue samples were incubated with primary antibodies overnight at 4 °C and with biotinylated secondary antibodies (The Jackson Laboratory, 1:500) for 3 hours at RT. Then, the slides were developed with diaminobenzidine (DAB, Dako), counterstained with hematoxylin, and visualized with an Olympus Dual-CCD microscope digital camera. Details and antibodies are listed in Supplementary Materials and Methods.

### Statistical analyses

The data were processed through the SPSS version 19.0 software (SPSS, Inc., Chicago, IL, USA). Quantitative data are expressed as the mean ± SD of at least three independent experiments. Continuous data were analyzed by Student’s t-test between two groups, whereas categorical data were analyzed by Mann-Whitney test. The association of RAD51-AS1 and RAD51 with clinicopathological features was assayed by χ^2^ test. Survival differences were assessed by the Kaplan–Meier method and log-rank test. Univariate and multivariate Cox regression analyses (Enter method) were performed to assess the relative risk for each factor. Correlation analysis of RAD51-AS1 with RAD51 and p53 was tested using Spearman correlation. All tests were two-sided, and P < 0.05 was considered significant.

## Electronic supplementary material

Supplementary Materials
