# Supplementary material for: E2F1-regulated long non-coding RNA RAD51-AS1 promotes cell cycle progression, inhibits apoptosis and predicts poor prognosis in epithelial ovarian cancer
Source: Sci Rep. 2017 Jun 30;7:4469. doi: 10.1038/s41598-017-04736-z (PMC5493660; doi:10.1038/s41598-017-04736-z)
Supplement: Supplementary file 1 — Supplementary Materials [file 41598_2017_4736_MOESM1_ESM.pdf]

Supplementary Materials

E2F1-regulated long non-coding RNA RAD51-AS1 promotes cell cycle progression, inhibits apoptosis and predicts poor prognosis in epithelial ovarian cancer

Xiaodan Zhang[1,2,3], Guoping Liu[4], Junjun Qiu[1,2,3], Ning Zhang[1,2,3], Jingxin Ding[1,2,3], Keqin Hua[1,2,3*]

[1]Department of Gynecology, Obstetrics and Gynecology Hospital of Fudan University, Shanghai, China

[2]Shanghai Medical College, Fudan University, Shanghai, China

[3]Shanghai Key Laboratory of Female Reproductive Endocrine-Related Diseases, Fudan University, Shanghai, China

[4]Institutes of Brain Science and State Key Laboratory of Medical Neurobiology, Fudan University, Shanghai, China

Corresponding author: Keqin Hua* (🖂)

🖂**Address:** Key Laboratory of Obstetrics and Gynecology Hospital of Fudan University, 413 Zhaozhou Road, Shanghai 200011, China. Phone: 86-15921515900, Fax: +86 21 63455090, Email address: keqinhua001@gmail.com

Supplementary Materials and Methods

1. Semi-quantitation of ISH signals

In brief, slides were heated and deparaffinized. Probe hybridization was performed for 2 hours at 40°C, followed by several rounds of amplification. DAB was used for signal detection and hematoxylin as counter stain. Technical and slide quality control was certified using a positive control probe targeting the common housekeeping gene PPIB and a negative control probe targeting the bacterial gene DapB (PPIB>2 and DapB<1). Finally, the slides were imaged using an Olympus Dual-CCD microscope digital camera, and semi-quantitative scores were generated utilizing the estimated number of punctate dots. ISH staining signals were categorized into five grades: 0, 1+, 2+, 3+ and 4+ according to the following table. In the tissue array, we harvested tissues from two sites for each tissue sample to reduce the influence of tumor heterogeneity and to be representative of the tumor. The average score was used to represent the expression levels of RAD51-AS1 on one tissue sample of an EOC patient.

**Staining score**	**Microscope objective scoring***
0	No staining or less than 1 dot to every 10 cells (40X magnification)
1	1–3 dots/cell (visible at 20–40X magnification)
2	4–10 dots/cell. Very few dot clusters (visible at 20–40X magnification)
3	>10 dots/cell. Less than 10% positive cells have dot clusters (visible at 20X magnification)
4	>10 dots/cell. More than 10% positive cells have dot clusters (visible at 20X magnification)

* Discount cells with artificially high nuclear background staining.

2. Quantitative real-time PCR

Total RNA was extracted using the TaKaRa MiniBEST Universal RNA Extraction Kit (Code No. 9767). Single-stranded cDNA was synthesized from 1 μg of RNA using the Thermo Scientific RevertAid First Strand cDNA Synthesis Kit (K1622). All amplifications and detections were performed using the Prism 7900 system (Applied Biosystems, Foster City, CA, USA) with SYBR Premix Ex Taq^{TM} (TaKaRa, RR420A). All reactions were performed according to the manufacturer's

instructions. Primers were synthesized by Sangon Biotech (Shanghai, China). Gene primers are shown below.

Gene	Sequence (5'to3')	Gene	Sequence (5'to3')
RAD51-AS1-	TACTGCCGAAACAAACCACA	CCNE2-	TCCCCAGCTTAAATCAGGCA
RAD51-AS1+	CCACGACTCCCAAGAGGTAA	CCNE2+	GGGGGATCAGTCCTTGCATT
GADD45B-	CGTGACCAGGAGACAATGC	CCNB2-	CTTCTTCCGGGAAACTGGCT
GADD45B+	GTGACAACGACATCAACATCG	CCNB2+	GGCTGGTACAAGTCCACTCC
FADD-	CCTCTTGTACCAGGTCAGCC	CCNB1-	ATGGCAGTGACACCAACCAG
FADD+	GGCTCGTCAGCTCAAAGTCT	CCNB1+	CCTACTGGGTCGGGAAGTCA
BNIP3L-	TAGCTCCACCCAGGAACTGT	GADD45A-	TTGATGTCGTTCTCGCAGCA
BNIP3L+	TCGTCCCACCTAGTCGAGC	GADD45A+	AGAGCAGAAGACCGAAAGCG
PTPN13-	TCCATTGACAGCTAGGACGC	CDKN1A-	GAAGGTAGAGCTTGGGCAGG
PTPN13+	GGGATAAGTGTCACGGGAGG	CDKN1A+	GCGACTGTGATGCGCTAATG
TNFRSF10B-	CCAGGTCGTTGTGAGCTTCT	CDKN2AIP-	TATCGGCACCCGAGGAAGAC
TNFRSF10B+	TGTCCACAAAGAATCAGGCATCA	CDKN2AIP+	CTAGCACGGATGAAGCTGCC
P53-	GCATTCTGGGAGCTTCATCT	CASP9-	GTCTTTCTGCTCGACATCACC
P53+	CTATGGAAACTACTTCCTGAAAACA	CASP9+	CGAACTAACAGGCAAGCAGC
PCNA-	CGCGTTATCTTCGGCCCTTA	CDK2-	GAAGATGGGGTACTGGCTTG
PCNA+	TCTGAGGGCTTCGACACCTA	CDK2+	ATGGACGGAGCTTGTTATCG
CDK1-	TCCTGCATAAGCACATCCTGA	IVD-	AGCTGGTTGATGCAGAGGTT
CDK1+	ACAGGTCAAGTGGTAGCCAT	IVD+	AGCAGAGGCAGGAATTTTGGA
CCNG1-	TGCAGTCATTCTGAGGCCAT	RAD51-	TTAGCTCCTTCTTTGGCGCA
CCNG1+	AGCTGAATGCCCTGTTGGAA	RAD51+	TGGAGCTAATGGCAATGCAGA
TYRO3-	ACTGGATGTCAGGCTCCTCCA		
TYRO3+	GCCGCCGCAGGTCTGA		

3. Details for ChIP experiment.

We designed primers for every 250-300bp in 3000bp upstream of RAD51-AS1 ORF. We used 10 primers totally.

Primers are as follows:

1	F agaggttggtcaacaggcaca	1-21
	R aaagtgctgggattacaggcg	268-288
2	F ccaaggcgggtggatcacca	295-314
	R ttttgacagacgattgtacat	551-571
3	F attattttcacaaccttggga	586-606
	R ctgggattacaggcatgtg	849-867
4	F cggtgagccaagattgtgcca	921-941

	R gcccattttgatatcttctgt	1191-1217
5	F gatgtccaacatcattattaa	1233-1253
	R acacctatgtaatcaccactt	1507-1527
6	F tgatcacagctcactgcagctt	1575-1596
	R cacaactgtatacaactccgtgaa	1824-1847
7	F ctacatgtatattacattgcgc	1861-1882
	R agtgtagtggcgcgatctcg	2155-2174
8	F ccagcctgggcggaagagcga	2175-2195
	R tttatgggttcgagcgagaagt	2415-2436
9	F gttgcctataaaggctctaat	2443-2463
	R ctggagagaggagcgctgc	2682-2700
10	F tccgacttcaccccgcgggc	2713-2732
	R gatccgggaggcggggatacg	2970-2990

Each primer sequences was checked to confirm that it would result in a ~300 bp product.

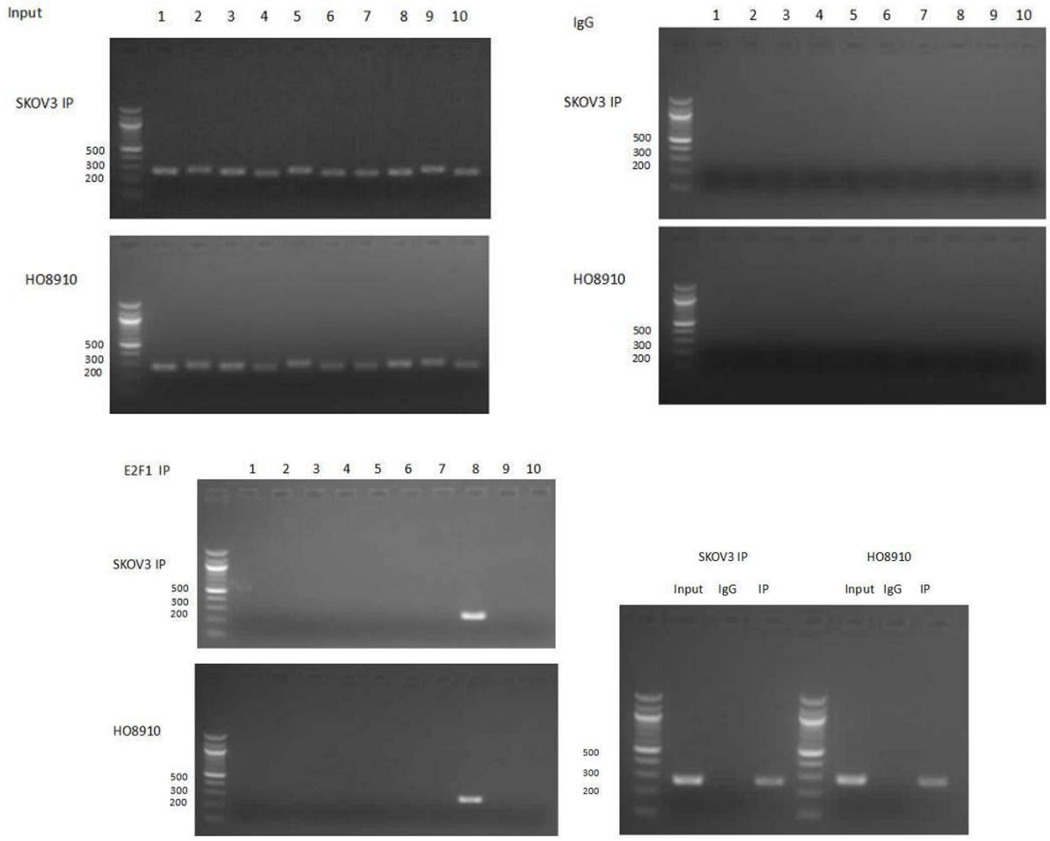

We found the putative binding site of E2F1 located in the NO.8 primer region, a sequence in RAD51 intron 1, at 564-825 bp upstream of the Open Reading Frame of RAD51-AS1. Then 722 - 730 bp upstream of the Open Reading Frame of RAD51-AS1 was mutated (TTTTCCGC →AATAGGCG) in the luciferase reporter experiments.

4. Plasmids used in Luciferase reporter experiments:

(1) E2F1 overexpression plasmid:

Vector： Lenti-EFS-GFP-WPRE; Element sequence： EFS-GFP-WPRE; Cloning site: AgeI/BamHI.

The following primers were used to amplify the E2F1:

F(E2F1-1) AgeI: cgACCGGTatggccttggccggggcc

R(E2F1-1) BamHI: cgGGATCCtcagaaatccaggggggtgaggtc

(2) RAD51-AS1 promoter plasmid:

Vector: PGL4.10; Element sequence： RAD51-AS1 promoter-Luc; Cloning site: KpnI; NheI

The following primers were used in PCR:

RAD51-AS1 F(KpnI): cgGGTACCcatgtatattacattgcgctttagaag

RAD51-AS1 R(NheI): cgGCTAGCagcggggagaaggcggatcc

(3) RAD51-AS1（mutation） promoter plasmid:

Vector: PGL4.10; Element sequence： RAD51-AS1（mutation） promoter-Luc; Cloning site: KpnI;NheI;

Mutation site(upstream 730-722bp of RAD51-AS1 ORF)：TTTTCCGC→AATAGGCG

The following primers were used in PCR for mutation and were confirmed by sequencing.

Mutation-1-F: tgaatggaataggcgattagatgatgaaaacactgttttg

Mutation-1-R: cgcctattccattcacatgatgcctag

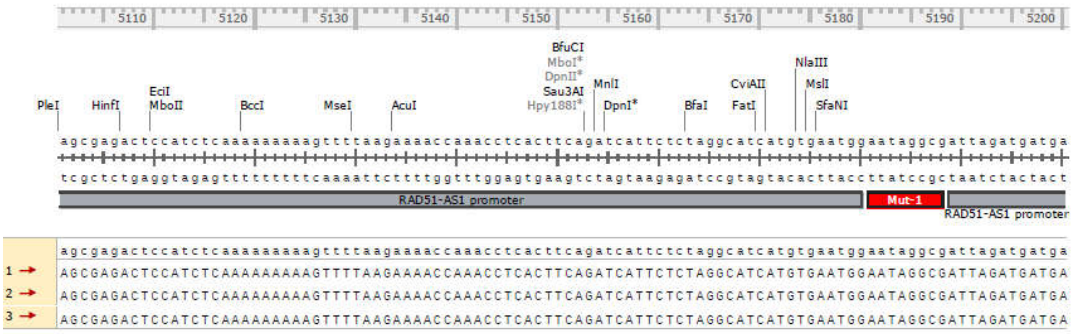

4. Primary Antibodies used in Western Blot analysis

anti-P53 (53kDa, Cell signaling Technology, 2527T, clone number 7F5, Rabbit IgG, 1:1000),

anti-CDKN1A (21kDa, Cell signaling Technology, 2947T, clone number 12D1, Rabbit IgG, 1:1000),

anti-CCNE2 (50kDa, Abcam, ab40890, clone number EP454Y, Rabbit IgG, 1:1000),

anti-FADD (23kDa, Abcam, ab108601, clone number EPR4415, Rabbit IgG, 1:1000),

anti-TNFRSF10B (47kDa, Abcam, ab181846, clone number EPR1695(2), Rabbit IgG, 1:10000),

anti-CASP3 (17/19kDa, Cell signaling Technology, 9661L, clone number Asp175, Rabbit IgG, 1:1000),

anti-CASP8 (55kDa, Abcam, ab108333, clone number EPR162, Rabbit IgG, 1:1000),

anti-CASP9 (46kDa, Abcam, ab32539, clone number E23, Rabbit IgG, 1:1000),

anti-Tyro3 (97kDa, Abcam, ab109231, clone number EPR4308, Rabbit IgG, 1:1000),

anti-IVD (46kDa, Abcam, ab123914, clone number 1B10, Mouse IgG, 1:1000),

anti-GAPDH (36kDa, Abcam, ab181602, clone number EPR16891, Rabbit IgG, 1:10000).

5. Xenograft tumors in nude mice

Sixteen engrafted mice were developed for tumor growth observation and randomly assigned to the RAD51-AS1-knockdown group or the NC group (8 mice per group). The engrafted mice were injected with ASOs targeting RAD51-AS1 or NC every 3 days to maintain the RNA-interference effect. The in vivo transfection complexes were formulated in the following proportions: 30 μl of transfection complexes=15 μg (15 μl) of oligonucleotides+7.5 μl of normal saline+7.5 μl of Entranster^{TM}–in vivo (Engreen Biosystem Co. China, Cat.No. 18668-11-1). All mice were injected with the same dose of transfection complexes at the same time, and the injections were performed as uniformly as possible. In addition, the injection dose increased over time. A starting dose of 30 μl was used in the first two weeks as the tumors were small, and then increased by 10 μl every 7 days. Tumor size and the signs of distress or changes in weight were observed every 3 days. Four weeks after the injection (when all tumors were <2.0 cm in diameter), the mice were sacrificed, and the tumors were weighed.

6. IHC details and antibodies

Tumor tissues were fixed with 4% paraformaldehyde (PFA) in phosphate-buffered saline (PBS) over night and then equilibrated in 30% sucrose in PBS and frozen in embedding medium (Tissue-Tek optimal cutting temperature compound; Sakura Finetek, Tokyo, Japan). Frozen sections of 12 μm were obtained using a cryostat (Microm; Leica, Wetzlar, Germany) and collected on Fisher Colorfrost-plus slides. Paraffin sections were used for the hematoxylin-eosin (H&E) staining of xenograft tumors in nude mice and for p53 in tissue array samples.

Primary antibodies were used as follows:

rabbit anti-Ki67 (1:500, Vector Laboratories, VP-K451);

rabbit anti-PH3 (1:500, Sigma-Aldrich, H0412, clone number $pSer^{10}$);

rabbit anti-Caspase3 (1:500, Cell signaling Technology, 9661L, clone number Asp175);

rabbit anti-Cyclin E2 (1:250, abcam, ab40890, clone number EP454Y);

rabbit anti-Caspase9 (1:250 , abcam, ab32539, clone number E23);

rabbit anti-P53 (1:150, Cell signaling Technology, 2527T, clone number 7F5).

rabbit anti-RAD51(1:500, abcam, ab133534, clone number EPR4030(3)).

Supplementary Figures

Figure. S1

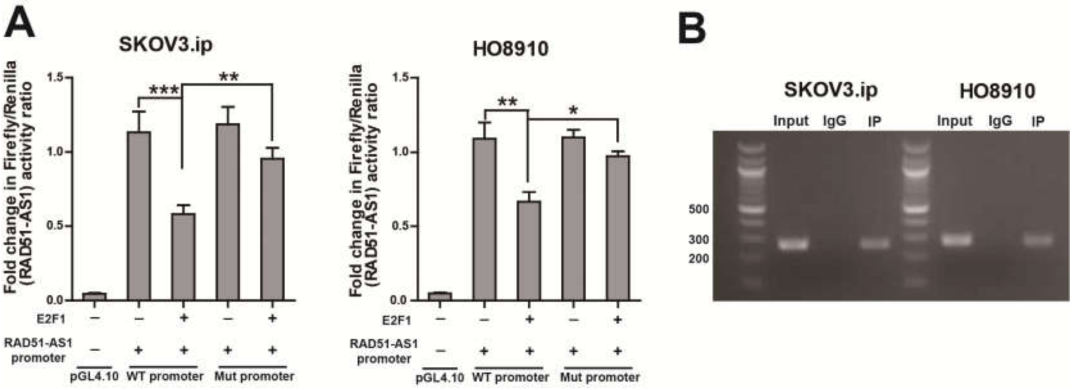

Figure. S1. RAD51-AS1 is negatively regulated by E2F1. A, E2F1 inhibited the transcription of RAD51-AS1 in dual-luciferase assays in SKOV3.ip and HO8910 cells. Mutation of the putative E2F1 binding site restored the inhibition of RAD51-AS1 promoter activity by E2F1. Each experiment was repeated in triplicate (n=3). (The results are shown as the mean±SD; Student's t test). **B**, E2F1 ChIP-PCR demonstrated E2F1 binding to RAD51-AS1 promoter in both SKOV3.ip and HO8910 cells.

Figure. S2

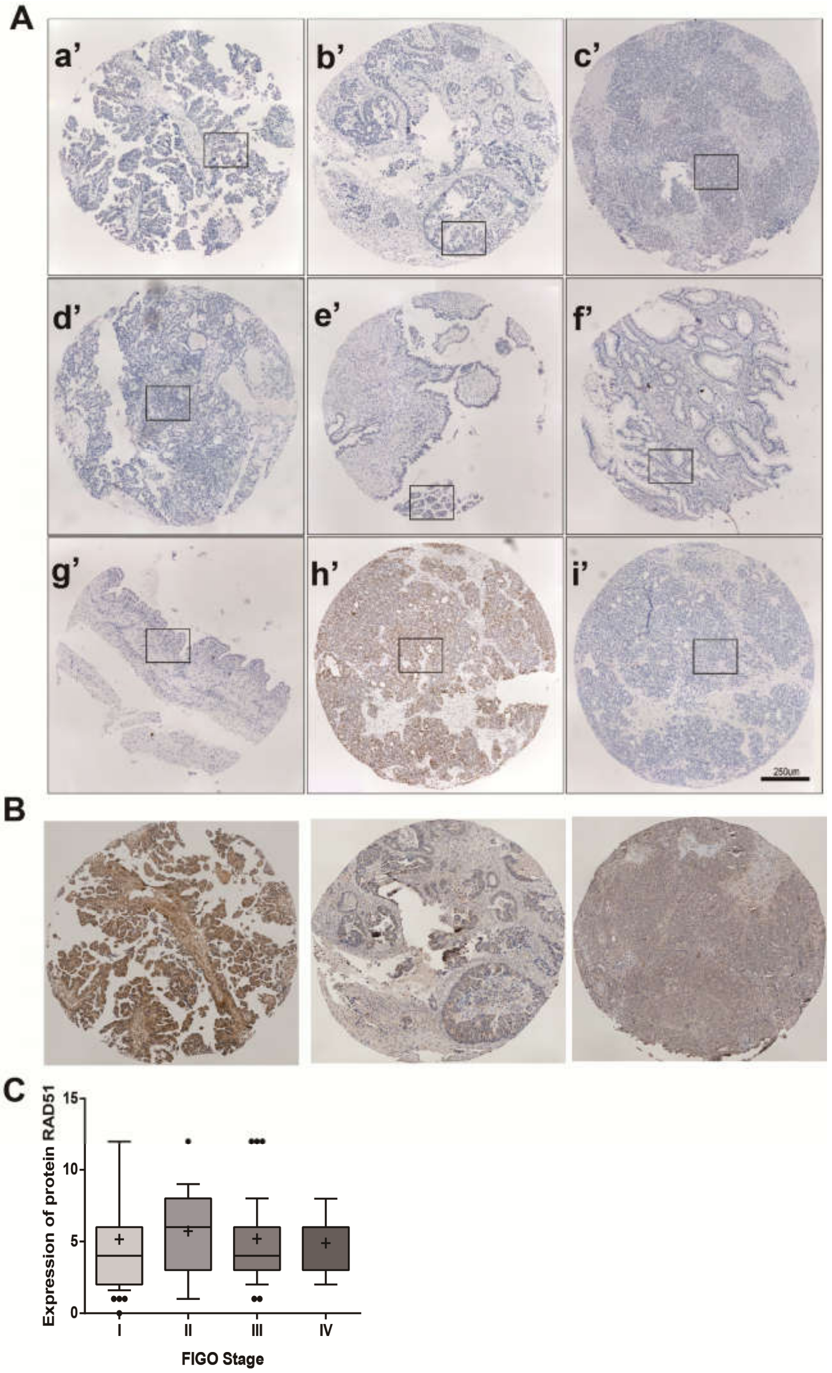

Figure. S2. Expression of RAD51-AS1 and RAD51 on the tissue microarrays. A, ISH of RAD51-AS1 on tissue microarrays. Pictures in Fig.1A are the magnification of the black boxed area in Supplementary Fig.S2A. Pictures a' - i' in Supplementary Fig.S2A correspond to the pictures a - i in Fig.1A, respectively. **B,** IHC of RAD51 on tissue microarrays. Pictures in Supplementary Fig.S2B correspond to the pictures a, b, c in Fig.S2A, respectively. **C,** RAD51 expression in different FIGO stages of EOC.

Figure. S3

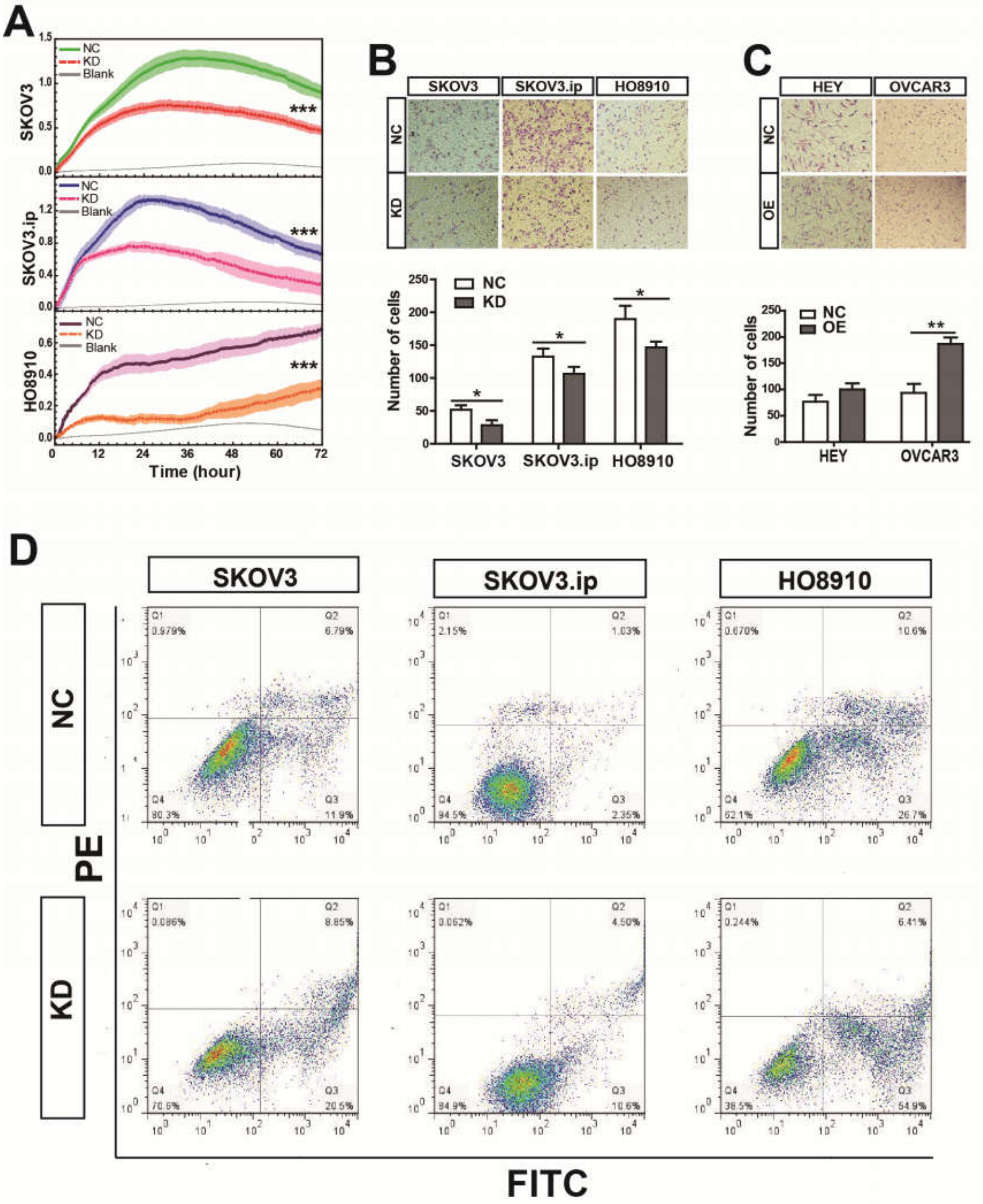

Figure. S3. Study of the function of RAD51-AS1 in cell migration and invasion with Mitomycin C added to the culture media to make the cells uniformly arrested. **A**, Migration assays. The real-time cell indexes indicated that RAD51-AS1 knockdown greatly decreased cell migration capacity compared with the NC cells. Blank control was performed with the bottom chamber containing RPMI 1640 but without FBS as a chemo-attractant. The light-colored error bars denote

the SD. **B**, Transwell invasion assays. RAD51-AS1 knockdown significantly decreased the invasive capacity of SKOV3, SKOV3.ip and HO8910 cells. **C,** Overexpression of RAD51-AS1 only increased cell invasion in the OVCAR3 cells, not in the Hey cells. **D,** Flow cytometric analysis of apoptosis. Each experiment was repeated in triplicate (n=3). (The results are shown as the mean±SD; *, $P<0.05$; **, $P<0.01$; ***, $P<0.001$ by Student's t test).

Supplementary Table S1

Supplementary Table S1-Association of RAD51 expression with clinicopathological features of EOC patients.

Variable	Low RAD51 expression (n=64)	High RAD51 expression (n=65)	P value
	n (%)	n (%)	
**Age (years)**			
<50	19 (29.7)	23 (35.4)	0.574
≥50	45 (70.3)	42 (64.6)	
**Histological subtype**			
Other	30 (46.9)	31 (47.7)	0.533
Serous	34 (53.1)	34 (52.3)	
**FIGO Stage**			
I-II	35 (54.7)	32 (49.2)	0.599
III-IV	29 (45.3)	33 (50.8)	
**Histological grade**			
Low	31 (48.4)	34 (52.3)	0.726
High	33 (51.6)	31 (47.7)	
**Tumor size (cm^3)**			
<1000	38 (59.4)	51 (78.5)	**0.023**
≥1000	26 (40.6)	14 (21.5)	
**Number of tumors**			
<2	28 (43.8)	32 (49.2)	0.598
≥2	36 (56.3)	33 (50.8)	
**Residual tumor diameter (cm)**			
<1	47 (73.4)	53 (81.5)	0.298
≥1	17 (26.6)	12 (18.5)	
**Lymph node metastasis**			
Absent	46 (71.9)	52 (80.0)	0.309
Present	18 (28.1)	13 (20.0)	
**Distant Metastasis**			
Absent	37 (57.8)	38 (58.5)	1.000
Present	27 (42.2)	27 (41.5)	
**CA125 level (U/ml)**			
<600	54 (84.4)	56 (86.2)	0.809
≥600	10 (15.6)	9 (13.8)	
**Ascites**			
Absent	48 (81.5)	48 (67.2)	1.000
Present	16 (18.5)	17 (32.8)	
**RAD51-AS1 level**			
Low	34 (53.1)	31 (47.7)	0.599
High	30 (46.9)	34 (52.3)	

NOTE: The median value of RAD51 expression was used as a cut-off for the low/high RAD51 expression groups. Data were analyzed using the χ^2 test. All tests were two-sided, and $P<0.05$ was considered significant.
